# Lack of association of two common polymorphisms on 9p21 with risk of coronary heart disease and myocardial infarction; results from a prospective cohort study

**DOI:** 10.1186/1741-7015-6-30

**Published:** 2008-10-16

**Authors:** Abbas Dehghan, Mandy van Hoek, Eric JG Sijbrands, Ben A Oostra, Albert Hofman, Cornelia M van Duijn, Jacqueline CM Witteman

**Affiliations:** 1 Department of Epidemiology, Erasmus Medical Center, Rotterdam, the Netherlands; 2 Department of Internal Medicine, Erasmus Medical Center, Rotterdam, the Netherlands; 3 Department of Clinical Genetics, Erasmus Medical Center, Rotterdam, the Netherlands

## Abstract

**Background:**

Recent genome wide association (GWA) studies identified two Single Nucleotide Polymorphisms (SNP) (rs10757278 and rs10757274) in the region of the CDK2NA and CDK2NB genes to be consistently associated with the risks of coronary heart disease (CHD) and myocardial infarction (MI). We examined the SNPs in relation to the risk of CHD and MI in a large population based study of elderly population.

**Methods:**

The Rotterdam Study is a population-based, prospective cohort study among 7983 participants aged 55 years and older. Associations of the polymorphisms with CHD and MI were assessed by use of Cox proportional hazards analyses.

**Results:**

In an additive model, the age and sex adjusted hazard ratios (HRs) (95% confidence interval) for CHD and MI were 1.03 (0.90, 1.18) and 0.94 (0.82, 1.08) per copy of the G allele of rs10757274. The corresponding HRs were 1.03 (0.90, 1.18) and 0.93 (0.81, 1.06) for the G allele of rs10757278. The association of the SNPs with CHD and MI was not significant in any of the subgroups of CHD risk factors.

**Conclusion:**

we were not able to show an association of the studied SNPs with risks of CHD and MI. This may be due to differences in genes involved in the occurrence of CHD in young and older people.

## Background

It has been considered for long that genes play a substantial role in susceptibility to coronary heart disease (CHD) [1]. Up to now, a limited number of these genes have been identified through the candidate gene approach and genome wide linkage studies. Recently a number of genome wide association (GWA) studies have identified several genetic variants on chromosome 9p21 associated with the risk of CHD. McPherson et al. found a Single Nucleotide Polymorphism (SNP), rs10757274, on chromosome 9p21 associated with the risk of CHD [2]. Helgadottir et al. found a close-by SNP, rs10757278, in the same 9p21 region associated with the risk of myocardial infarction (MI) [3]. These findings were followed by another GWA study by Samani et al [4], which found rs1333049 to be associated with the risk of coronary artery disease [4]. All three SNPs are located within the same Linkage Disequilibrium (LD) block on chromosome 9 approximately 22 million base pairs from the 9p telomere, adjacent to two tumor suppressor genes, CDKN2A and CDKN2B. These genes are involved in regulation of cell proliferation. Abnormal proliferation is one of the characteristics of atherosclerosis, one of the pathological features of CHD and MI [5].

To date, the findings are replicated in several case-control studies comprising 12285 cases and 23184 controls and two cohort studies comprising 22056 subjects [6-10]. These replications has made this locus one of the best replicated findings for genetic susceptibility to cardiovascular diseases. Though this findings are promising, they will be of more clinical worth if translated to older patients who constitute a larger part of the patients. We chose to study rs10757278 and rs10757274 because they were most strongly and consistently associated with CHD and MI in GWA studies. The leading SNP of the study by Samani et al, rs1333049, is in the same LD block with rs10757278 and contributes to the same haplotype alleles. We attempted to replicate the association in the Rotterdam Study, a population-based cohort study among older subjects, but found no association.

## Methods

### Study Population

The study was conducted within the framework of the Rotterdam Study, an ongoing prospective, population-based cohort study on determinants of a number of chronic diseases. The Rotterdam Study has been described in detail elsewhere [11]. In brief, all inhabitants of Ommoord, a district of Rotterdam in the Netherlands, who were 55 years or over, were invited to participate in this study. Of all 10275 eligible individuals, 7983 agreed to participate (78%). Written informed consent was obtained from all participants and the Medical Ethics Committee of the Erasmus Medical Center approved the study.

### Baseline measurements

The baseline examinations took place from 1990–1993. Participants were visited at home for an interview. Information on current health status, medical history, use of medication, and smoking status were obtained during the interview. The interview was followed by two visits at the research center for blood sampling and further examinations.

At baseline, participants were asked whether they have ever experienced a heart attack. A 12-lead electrocardiogram (ECG) was stored digitally and analyzed by using the Modular ECG Analysis System (MEANS). Myocardial infarction found on ECG was based on criteria partly derived from the Minnesota code. A history of myocardial infarction was considered present in case of a self-report of myocardial infarction confirmed by ECG or additional clinical information, or the presence of an ECG characteristic of prior myocardial infarction [12,13].

### Genotyping

Genomic DNA was extracted from leucocytes following standard procedures. Participants were genotyped for rs10757274 and rs10757278. Genotypes were determined in our study population in 2-ng genomic DNA by use of pre-designed TaqMan SNP genotyping assay (Assay ID C__26505812_10 and C__11841860_10, respectively; Applied Biosystems, Foster City, CA). Reactions were performed with the Taqman Prism 7900 HT 384 wells format.

### Follow up

Follow up for clinical events started at baseline and follow up examinations were carried out periodically in 1995–1996, 1997–1999, and 2002–2004. Participants were continuously monitored for fatal and nonfatal cardiovascular events through automated linkage with files from general practitioners and pharmacies working in the study district of Ommoord. In addition, all medical records of the participants under the care of general practitioners outside the study area were checked annually. Two research physicians independently coded all reported events according to the International Classification of Diseases, 10th edition (ICD-10). Codes on which the research physicians disagreed were discussed to reach consensus. Finally, a medical expert in cardiovascular disease, whose judgment was considered final, reviewed all events. Information on vital status was obtained regularly from municipal health authorities in Rotterdam. For the present study, follow up data were available until October 1, 2005.

### Incident coronary heart disease and myocardial infarction

To identifying incident myocardial infarction and coronary heart disease, we collected information from baseline (1990 – 1993) until January 1, 2005. Fatal or non-fatal MI reported by general practitioners in the research area, letters from medical specialists and discharge reports for hospitalized patients were the sources of information used. Two research physicians coded the events independently and in case of disagreement the consensus was made ina separate session. Finally a specialist whose judgment was considered final verified the coding. We defined incident MI as fatal or non fatal MI (ICD-10 code I21). Incident CHD was defined as fatal or nonfatal myocardial infarction (ICD-10 code I21), coronary artery bypass grafting (CABG), and percutaneous transluminal coronary angioplasty (PTCA).

### Population for analysis

The SNPs, rs10757274 and rs10757278 were genotyped in 6251 and 6265 out of 7129 participants who visited the research center at baseline.

### Statistical analysis

Genotype frequencies were tested for Hardy-Weinberg equilibrium with a chi-square test using The Hardy-Weinberg package for R . To compare the baseline characteristics between healthy subjects and those who experienced CHD or MI, we used chi-square for categorical variables and ANOVA for continuous variables.

A Cox regression analysis was used to assess the association of SNPs with incident CHD and MI. The proportional hazards assumption was validated by the use of a time-dependent variable to check increasing or decreasing trends in the hazard ratio (HR) over time. The basic model was adjusted for age and sex. The multivariate adjusted model was additionally adjusted for BMI, systolic and diastolic blood pressure, total cholesterol, HDL cholesterol, smoking, and diabetes.

To examine whether the effect of SNPs vary by the level of other risk factors we performed the analysis stratified by age, sex, family history of cardiovascular disease, HDL cholesterol, diabetes, hypertension, smoking, and history of CHD. For smoking, participants were categorized to never, former, and current smokers. For hypertension and diabetes, participants were categorized into those with and without the condition. History of CHD was defined as a history of MI, percutaneous transluminal coronary angioplasty (PTCA) or coronary artery bypass grafting (CABG). For other risk factors, the population was divided into two equal subgroups by use of the median. All statistical analyses were performed with the use of SAS, version 8.

## Results

Table 1 shows the baseline characteristics of the studied population. CHD and MI cases were significantly older, and more often male, hypertensive, diabetic, and smoker than subjects without these conditions. Moreover, systolic blood pressure, and total cholesterol were significantly higher and HDL cholesterol was significantly lower in CHD and MI cases. We found none of these characteristics to be significantly associated with the studied SNPs.

**Table 1:** Baseline characteristics of all participants, and of cases with incident MI and incident CHD

Characteristics	All participants	MI cases	P value	CHD cases	P value
Number	6251	412	-	588	-
Age, mean (SD), y	69.5 (9.1)	70.3 (7.8)	0.02	68.6 (7.4)	0.23
Men, (%)	40.4	58.7	<0.001	61.2	<0.001
Body mass index, mean (SD), kg/m2	26.3 (3.7)	26.3 (3.5)	0.29	26.3 (3.4)	0.07
Waist circumference, mean (SD), cm	90.5 (11.2)	91.9 (10.0)	0.89	92.2 (10.2)	0.33
Systolic blood pressure, mean (SD), mm Hg	139.3 (22.2)	142.3 (21.9)	0.01	141.3 (21.7)	0.004
Diastolic blood pressure, mean (SD), mm Hg	73.7 (11.5)	73.7 (11.5)	0.77	73.7 (11.3)	0.38
Hypertension, (%)	34.3	40.6	0.006	40.2	0.002
Diabetes, (%)	10.5	17.0	<0.001	15.8	<0.001
Total cholesterol, mean (SD), mmol/L	6.6 (1.2)	6.9 (1.2)	<0.001	6.9 (1.2)	<0.001
HDL cholesterol, mean (SD), mmol/L	1.3 (0.4)	1.2 (0.3)	<0.001	1.2 (0.3)	<0.001
Current smoker, (%)	22.8	26.0	<0.001	27.1	<0.001
Former smoker, (%)	41.5	49.5		51.5	

During follow-up, 588 participants developed CHD, of which 412 had experienced an MI. The mean follow-up time was 9.5 years for CHD (incidence rate 9.9 per 1000 person years) and 9.5 years for MI (incidence rate 6.9 per 1000 person years). Mean (standard deviation) age of onset was 68.6 (7.4) for CHD and 70.3 (7.8) years for MI. We examined the associations of rs10757274 and rs10757278 with risks of CHD and MI (table 2, 3 and 4). None of the SNPs were significantly associated with the risk of CHD or MI. The age and sex adjusted HR (95% confidence interval [CI]) for CHD and MI were 1.03 (0.90, 1.18) and 0.94 (0.82, 1.08) per copy of G allele of rs10757274, respectively. The corresponding HRs were 1.03 (0.90, 1.18) and 0.93 (0.81, 1.06) per copy of G allele of rs10757278.

**Table 2:** The age and sex adjusted and multivariate adjusted association of the SNPs with incident CHD and MI

		Allele	All participants	Incident cases	Age and sex adjusted	Multivariate adjusted HR*
CHD	rs10757274	G	45.8	45.5	1.03 (0.90, 1.18)	1.00 (0.87, 1.15)
	rs10757278	G	44.9	42.7	1.03 (0.90, 1.18)	1.00 (0.87, 1.15)
MI	rs10757274	G	45.8	43.8	0.94 (0.82, 1.08)	0.97 (0.84, 1.11)
	rs10757278	G	44.9	42.7	0.93 (0.81, 1.06)	0.95 (0.83, 1.10)

* adjusted for age, sex, BMI, serum total and HDL cholesterol, smoking, diabetes, systolic and diastolic blood pressure.

CI = Confidence interval, CHD = Coronary heart disease, MI = Myocardial infarction

**Table 3:** The age and sex adjusted and multivariate adjusted association of the SNPs with incident CHD

	Participants (cases)	Age and sex adjusted	Multivariate adjusted *
rs10757274			
AA	1834 (184)	Reference	Reference
AG	3107 (273)	0.89 (0.74, 1.07)	0.90 (0.74, 1.09)
GG	1310 (131)	1.00 (0.80, 1.26)	0.99 (0.78, 1.25)
			
rs10757278			
AA	1909 (188)	Reference	Reference
AG	3097 (269)	0.90 (0.74, 1.08)	0.90 (0.74, 1.09)
GG	1264 (127)	1.01 (0.81, 1.27)	1.00 (0.79, 1.26)

* adjusted for age, sex, BMI, serum total and HDL cholesterol, smoking, diabetes, systolic and diastolic blood pressure.

CI = Confidence interval, CHD = Coronary heart disease, MI = Myocardial infarction

**Table 4:** The age and sex adjusted and multivariate adjusted association of the SNPs with incident MI

	Participants (cases)	Age and sex adjusted	Multivariate adjusted *
rs10757274			
AA	1832 (133)	Reference	Reference
AG	3106 (197)	0.90 (0.72, 1.12)	0.94 (0.75, 1.19)
GG	1309 (82)	0.89 (0.67, 1.17)	0.94 (0.70, 1.25)
			
rs10757278			
AA	1907 (139)	Reference	Reference
AG	3096 (187)	0.85 (0.68, 1.06)	0.89 (0.71, 1.12)
GG	1263 (80)	0.88 (0.67, 1.16)	0.92 (0.70, 1.23)

* adjusted for age, sex, BMI, serum total and HDL cholesterol, smoking, diabetes, systolic and diastolic blood pressure.

CI = Confidence interval, CHD = Coronary heart disease, MI = Myocardial infarction

We repeated the analysis with incident cases limited to those occurred before age 70. Age and sex adjusted HR (95% CI) for CHD and MI were 1.00 (0.97, 1.04) and 0.90 (0.74, 1.09) per copy of G allele of rs10757274. The corresponding HRs for MI were 1.00 (0.96, 1.04), and 0.91 (0.75, 1.10) per copy of G allele of rs10757278.

**Figure 1:**
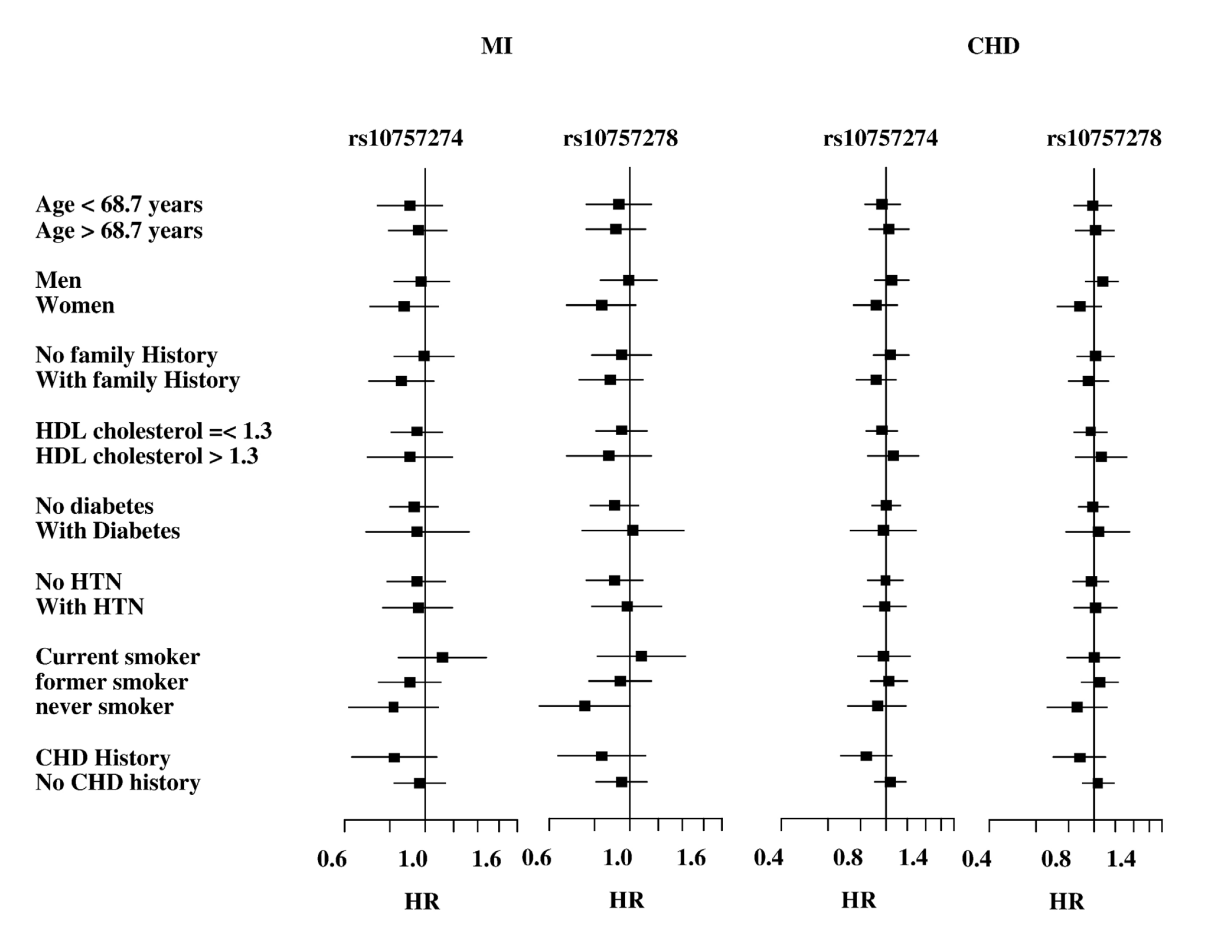
**Association of rs10757274 and rs10757278 with CHD and MI in subgroups of cardiovascular risk factors.** The solid squares centered are on the hazard ratio estimate and scaled proportional to the sample size. Horizontal bars show the 95% confidence interval.

To investigate whether any of the covariates affect the relation of SNPs with CHD and MI, we repeated the analysis in subgroups of age, sex, family history of cardiovascular disease, HDL cholesterol, diabetes, hypertension, smoking, and history of CHD (figure 1). The association was not significant in any of the studied subgroups and no significant interaction was found.

## Discussion

Our main goal was to replicate the results of recent GWA studies on CHD and MI in a population based study. Two SNPs, rs10757274 and rs10757278, which were most consistently and strongly associated with the risk of CHD in GWA studies were studied. We not only found no significant association between these two SNPs and the risks of CHD and MI, but also found, however non-significant, an inverse direction for the risk. We also did not find an association in subgroups of cardiovascular risk factors.

Different approaches have been used in recent decades to discover causal genes for cardiovascular diseases. A novel approach is the GWA study which searches large part of the genome for predisposing variants. Contrary to the formerly common approach, the candidate gene study, the GWA study is a hypothesis free approach, i.e. it holds no prior assumption on the location of predisposing genes. As an advantage, this approach promises a more comprehensive understanding of the causal genes. However, this method is liable to false positive findings. Hence, GWA studies always need to be replicated in independent samples to confirm their findings.

Our study had sufficient power to detect effect sizes as shown in the published studies. In an additive model and for a SNP with a minor allele frequency of 0.45 (lowest minor allele frequency: 0.43, shown in Icelandic population A [3]), our study had more than 80% power to detect a relative risk of 1.15 for CHD (the lowest effect: 1.18, shown in ARIC study [2]) and 1.23 for MI (the lowest effect: 1.25, shown in the Iceland population A) [14].

We did not find any of the SNPs to be associated with the risk of CHD in our study. One legitimate conjecture for the inconsistency of our results with former studies may be heterogeneity of the effect. Compared to the Rotterdam Study, most of the studied populations comprised young CHD or MI patients. If the risk allele on chromosome 9p21 invokes only early onset of CHD, the effect in older subjects may not be large enough to be found in our study. Therefore, our negative finding may point to a heterogeneity of effect by age. In agreement with this conjecture, Helgadottir et al. showed that the association was stronger when only those with early onset MI were considered [3]. However, we failed to find an evidence of age affecting the association in our data. The strength of the association did not change materially when we limited the incident cases to those developed CHD or MI before age 70. Moreover, the strength of the association was not significantly different in age subgroups (figure 1). We emphasis that our study may be underpowered to detect the effect of age on the association. It is noteworthy that the heterogeneity of effect has a particular clinical and public health impact. In Western countries the majority of morbidity and mortality from CHD occurs in elderly people. In the Netherlands, 74% of men and 91% women who experience fatal MI are older than 65 years . The fact that CHD is less common in younger population implies that these subjects are not a good representative of the general population of CHD patients. Therefore, caution should be taken in generalizing the results of the published studies to an elderly population.

One may also speculate that those carrying the risk allele had developed CHD at early age and were excluded at the baseline in our study. If this is true, the prevalence of risk allele should be higher in those who had a history of CHD at baseline i.e. prevalent cases of CHD. To examine this issue, we studied the association of the SNPs with prevalent CHD cases but found no association (data not shown). Moreover, the frequencies of the alleles in our population were high and comparable to former studies making selection bias unlikely.

Previous studies mainly employed standard case-control association studies. Our study has the advantage of employing a different approach, the prospective study in a large population based sample. One potential limitation of our study is that the participants were not fully followed and healthy subjects who carry the risk allele may later develop the disease.

## Conclusion

In conclusion, we showed that the studied SNPs are not major players in development of CHD in the elderly population. Our negative finding offer a new perspective on 9p21 SNPs and shows that the association does not hold for all CHD cases. The lack of association may be due to the difference in genes involved in the development of CHD in young and older people. Individualized preventive measures and therapies constitute a major long-term goal of GWA studies. Heterogeneity of the effect, therefore, has substantial public health impact and needs to be acknowledged.

## Abbreviations

GWA: Genome Wide Association; SNP: Single Nucleotide Polymorphism; CHD: Coronary Heart Disease; MI: Myocardial Infarction; HR: Hazard Ratio; LD: Linkage Disequilibrium; ECG: Electrocardiogram; ICD: International Classification of Diseases; CABG: Coronary Artery Bypass Grafting; PTCA: Percutaneous Transluminal Coronary Angioplasty.

## Competing interests

The authors declare that they have no competing interests.

## Authors' contributions

AD contributed to the design of this study, performed the statistical analysis, and drafted the manuscript. MvH, EJGS, and JCMW contributed to the conception and the design of this study and critically revised the manuscript. AH, CvD, and BO has made substantial contributions to the interpretation of results, and critically revised the manuscript. All authors have read and approved the final manuscript.

## Pre-publication history

The pre-publication history for this paper can be accessed here:


